# Community-based mechanisms underlying the root cadmium uptake regulated by Cd-tolerant strains in rice (*Oryza sativa*. L)

**DOI:** 10.3389/fpls.2023.1196130

**Published:** 2023-08-11

**Authors:** Peng Li, Ziqin Xiong, Yunhe Tian, Zhongyi Zheng, Zhixuan Liu, Ruiwen Hu, Qiming Wang, Hejun Ao, Zhenxie Yi, Juan Li

**Affiliations:** ^1^ College of Agronomy, Hunan Agricultural University, Changsha, China; ^2^ Hunan Rice Research Institute, Hunan Academy of Agricultural Sciences, Changsha, China

**Keywords:** rice, cadmium pollution, microbiological control, endophytic bacterial community, interaction

## Abstract

In recent years, the problem of Cd pollution in paddy fields has become more and more serious, which seriously threatens the safe production of food crops and human health. Using microorganisms to reduce cadmium pollution in rice fields is a green, safe and efficient method, the complicated interactions between the microbes in rice roots throughout the process of cadmium absorption by rice roots are poorly understood. In this investigation, a hydroponic pot experiment was used to examine the effects of bacteria R3 (*Herbaspirillum* sp) and T4 (*Bacillus cereus*) on cadmium uptake and the endophytic bacterial community in rice roots. The results showed that compared with CK (Uninoculated bacterial liquid), the two strains had significant inhibitory or promotive effects on cadmium uptake in rice plant, respectively. Among them, the decrease of cadmium content in rice plants by R3 strain reached 78.57-79.39%, and the increase of cadmium content in rice plants by T4 strain reached 140.49-158.19%. Further investigation showed that the cadmium content and root cadmium enrichment coefficient of rice plants were significantly negatively correlated with the relative abundances of *Burkholderia* and *Acidovorax*, and significantly positively correlated with the relative abundances of *Achromobacter*, *Agromyces* and *Acidocella*. Moreover, a more complex network of microbes in rice roots inhibited rice plants from absorbing cadmium. These results suggest that cadmium uptake by rice plants is closely related to the endophytic bacterial community of roots. This study provides a reference scheme for the safe production of crops in cadmium contaminated paddies and lays a solid theoretical foundation for subsequent field applications.

## 1 Introduction

Cadmium (Cd) is not essential to plant growth, but is toxic to plants (Huang et al., 2020). When a specific concentration is reached, it hurts plant cell structure and disrupts normal cell physiological processes, and lead to abnormal growth and development of plants or death (Wan and Zhang, 2012). Further entry into people and animals via the food chain will seriously threaten their life and health (Kukusamude et al., 2021). As a significant food crop, rice (*Oryza sativa* L.) is one of the main sources of Cd buildup in the human body (Li et al., 2017). A better understanding of the mechanism of Cd uptake and accumulation in rice is essential for developing strategies to prevent excessive Cd from entering the food chain. Plant roots are the most critical site for Cd absorption and accumulation (Song et al., 2016). Therefore, it is crucial to comprehend the process by which Cd is absorbed by rice roots. The employment of microorganisms to control Cd absorption by crops has sparked a lot of attention due to its low cost, significant effect, and ecological environmental protection (Cheng et al., 2021b; Wang et al., 2022). At present, numerous research has revealed that adding strains to rice’s rhizosphere not only helps the plant grow and develop (Kuang et al., 2016), but also increases the plant’s ability to withstand Cd while lowering its Cd content (Ma et al., 2016; El-Esawi et al., 2020; Cheng et al., 2021a).

Microbial control of Cd pollution in rice is mainly achieved through two aspects. First, microorganisms can interact with Cd in the environment to passivate or chelate Cd ions, as a result, the bioavailability of Cd in the environment is reduced, increasing rice tolerance to Cd and reducing Cd absorption by roots to meet the goal of maintaining normal rice development and expansion and lowering the Cd content of rice grains (Xu et al., 2018; Zhang et al., 2018; Cui et al., 2020; Li et al., 2021). Meanwhile, microorganisms can also secrete organic acids that release Cd into ionic form, increasing its bioavailability and promote its absorption by plants (Chen et al., 2014; He et al., 2020), thereby reducing the Cd content in paddy fields. Microorganisms with this latter function are often used in conjunction with other crops before rice production to reduce Cd content in the paddy field, to achieve the goal of lowering the Cd content of rice when replanting rice. Second, microorganisms can directly interact with plants to influence the expression of key channel proteins involved in Cd uptake and transport, hence influencing Cd uptake and transport in plants (Chatterjee et al., 2020; Zhou et al., 2020). Furthermore, the structure and function of the plant endophytic bacterial community are linked to Cd uptake and transport (Teixeira et al., 2014). The structure and function of this community will change according to the interaction between exogenous endophytic bacteria and their own endophytic bacteria, thus either limiting or boosting Cd absorption and transport by plants (He et al., 2022).

In this study, we used Cd-tolerant endophytic bacteria previously isolated from rice seeds in the laboratory and selected two strains with strong comprehensive physiological functions for rice hydroponic pot experiments, to investigate the impact of inoculation on Cd absorption in rice and analyze the microbial community of rice roots after inoculation. Overall, the goal of this research is to: (1) Analyze the growth and Cd accumulation of rice plants at the highest tillering stage after inoculation with abovementioned strains; (2) Explore the effect of endophytic bacterial inoculation on the endophytic bacterial community in the roots of rice plants; (3) Elucidate the possible mechanism(s) by which inoculation of endophytic bacteria affect Cd uptake in rice plants. This work offers a new method for controlling Cd pollution in rice fields, reducing Cd buildup in rice grains, and a new practical value for the utilization of moderately and mildly Cd-contaminated farmland. It is of great significance for mitigating environment problems and ensuring food security.

## 2 Materials and methods

### 2.1 Experiment material

The tested rice (*Oryza sativa*. L Huanghuazhan), a thermosensitive conventional rice variety that readily accumulates Cd, was provided by the Hunan Rice Research Institute. The water sorting method was used to screen out the full-grained rice seeds, after that, the seeds were disinfected by soaking them in a 5% solution of sodium hypochlorite for 15 minutes and then rinsing them 8-10 times with purified water to make sure that no sodium hypochlorite solution remained. After soaking the sterilized seeds for 2-3 days in clean water, they were laid flat in a germination box and incubated in a constant temperature incubator (30°C) until they reached the three-leaf stage. Rice seedlings with identical development and size were chosen for pot experiments.

Cd-resistant endophytic bacterial strains R3 (*Herbaspirillum* sp) and T4 (*Bacillus cereus*) were screened from Huanghuazhan rice seeds in a previous study. All organisms stocks were stored at -80°C. The two strains were seeded into 500 mL of LB liquid solution at 5% before the test and shaken for 36 hours at 30°C and 180 rpm. Low-temperature high-speed centrifugation was used to remove the fermentation broth. The bacteria were resuspended in 0.85% KCl solution, the cell counting plate was used to determine the concentration under the optical microscope, and 1 × 10^8^ cfu·mL^-1^ bacterial solution was prepared for use. The nutrient solution tested was Kimura B (Yue et al., 2015), and exogenous Cd was added as CdCl_2_ to provide a nutritional solution with a Cd content of 0.3 mg·L^-1^ (Simulate farmland with moderate cadmium pollution in China).

### 2.2 Pot experiment design

Rice hydroponic pot trials were conducted in a greenhouse at Hunan Agricultural University in April 2017, with daytime temperatures of 29-40°C and nighttime temperatures of 24-29°C, and a relative humidity of 65-85%. The pot was a polyethylene (PE) cuboid basin with an inner length of 65 cm, a width of 40 cm, and a height of 16 cm. Each basin was divided into 20 L Kimura B nutrient solution (Cd concentration 0.3 mg·L^-1^, pH 7.0). The rice seedlings were transplanted into hydroponic pots and fixed with foam plates and planting baskets, after one week of culture, the strains were inoculated on the roots of the rice seedlings (100 mL 1×10^8^ cfu·mL^-1^ bacterial suspension). Three treatment groups were established: (1) R3: Inoculate R3 strain; (2) T4: Inoculate T4 strain; (3) CK: The blank control group was not inoculated with strain, and the same amount of distilled water was added. For each treatment, eight replicate pots were put up. During the rice growing time, the rice was cultured to the highest tillering stage by regularly adding the same amount of nutrient solution. Random sampling was used to measure the dry matter weight of rice plants and the Cd content of various plant components, and further analysis was done on changes in the endophytic microbial community of rice roots.

### 2.3 Sampling

For each treatment, random sampling was done when the rice was at its highest tillering stage, 8 plants was used to detect dry matter weight and cadmium content, and 4 plants was used to detect microbial community. The entire rice plant was split into roots and aboveground components, then thoroughly cleaned using distilled water, packed into a clean envelope bag, respectively, placed in an oven at 105°C for 30 minutes, dried to a constant weight at 80°C, and then the dry weight of each portion was measured. Each sample was then pulverized into a powder, and after further processing, the Cd content of each part of the plant was detected.

For surface sterilization and disinfection, the rice roots of each treatment were first washing for three minutes in 5% NaClO after being rinsed with pure water, followed by 5 minutes in 75% ethanol. Finally, the roots were repeatedly rinsed with pure water to remove any remaining chemicals from the root surface. The roots were pounded into a powder in a sterile mortar using liquid nitrogen for subsequent DNA extraction.

### 2.4 Detection of biomass and Cd content

Rice roots and aboveground sections were weighed using an electronic balance to assess their dry matter content. 0.5 g samples of various sections of the rice plant were weighed into polytetrafluoroethylene jars, to which 5 mL of premium pure nitric acid (GR, 99.7%) were added, after which the jars were sealed and left overnight. Next morning 1 mL of H_2_O_2_ was added and the samples then placed in a microwave digestion apparatus (Changzhou, DTD-50). After the digestion was complete, it was allowed to cool, before slowly opening the tank cover to exhaust. The digestion tank was placed on a temperature-controlled electric hot plate at 150 °C to reduce the acid concentration in the solution. After cooling again, after being transferred to a 10.0 mL volumetric flask, the digestion solution, and the digestion tank was washed 3 times with a small amount of distilled water, combine the washings in the volumetric flask (50 mL) and diluted to the mark, and mixed well. By using inductively coupled plasma mass spectrometry (ICP-MS, EXPEC 7200, China), the Cd concentration was measured (Peng et al., 2014).

### 2.5 Detection of endogenous microbial communities

The Plant Genome DNA Extraction Kit (TransGen Biotech) was used to extract DNA from various parts of the rice plant, and the extracted genomic DNA was used as a template for PCR amplification. The V5-V7 region was selected for amplification of 16S rRNA gene using specific primers 799 F (5 ‘-AACmGGattagataccCGG-3’) and 1115 R (5 ‘-AGGGTTGCGCTCGTTG-3’) (Kembel et al., 2014). The length and concentration of PCR amplification products were detected by 1% agarose-gel electrophoresis. After qualified detection, a library was constructed. Finally, PE250 sequencing was performed on the constructed amplified library using Illumina Nova 6000 platform.

### 2.6 Data processing and statistical analysis

The data were processed under the guidance of Zheng (Zheng et al., 2022), as detailed in the **Supplementary Material**.

## 3 Results

### 3.1 Effects of endophytic bacteria on dry matter weight and Cd uptake and transport in rice plants

The strains were inoculated after transplanting of the three-leaf period of rice seedlings, and they were cultured until the plants reached their highest tillering stage. The action time of the strains was 24 days. According to **Table 1**, compared to CK, the inoculation of strains R3 and T4 had no significant effect on the dry matter weight of the roots and aboveground portions of rice plants (*P* < 0.05).

**Table 1:** Effects of endophytic bacteria on dry matter weight and Cd uptake in rice plants.

Treatment	Dry matter weight/g·plant^-1^		Cd concentration/mg·kg^-1^		BCF	TF
	Root	Aboveground part	Root	Aboveground part		
CK	0.66 ± 0.05a	2.59 ± 0.16a	28.01 ± 1.31b	2.28 ± 0.27b	93.36 ± 4.36b	0.082 ± 0.012a
R3	0.64 ± 0.04a	2.44 ± 0.17a	6.08 ± 0.81c	0.47 ± 0.09c	20.25 ± 2.72c	0.079 ± 0.022a
T4	0.63 ± 0.02a	2.52 ± 0.21a	72.32 ± 7.21a	5.49 ± 0.74a	241.01 ± 24.04a	0.076 ± 0.008a

According to the Duncan test, mean values (± S.D., n = 8) with distinct letters differ substantially between CK, R3 and T4 treatments (P < 0.05). BCF of rice root to Cd in Nutrient solution, TF of Cd from rice roots to aboveground parts.

**Table 1** shows that for each treatment, the roots of the rice plants had a higher Cd content than the aboveground sections. However, the Cd uptake of rice plants showed two divergent trends, promotion and inhibition, with the inoculation of the different strains. Inoculation with strain R3 may dramatically lower the Cd content of rice roots and aboveground portions when compared to CK (*P* < 0.05), with reduction rates reaching 78.29% and 79.36%, respectively, while the Cd content of rice roots and aboveground sections may be greatly increased by inoculation with strain T4 (*P* < 0.05), at rates of 158.19% and 140.79%, respectively. When rice is in its highest tillering stage, compared with CK, Cd accumulation in roots and aboveground parts of rice plants treated with both strains changed significantly (*P* < 0.05), and Cd was mainly enriched in the roots of rice plants (**Table S1**). Among them, the inoculation of R3 strain significantly reduced the cadmium accumulation in the roots and aboveground parts of rice plants, and the inoculation of T4 strain significantly increased the cadmium accumulation in the roots and aboveground parts of rice plants (*P* < 0.05). In comparison to CK, inoculation with strain R3 considerably reduced and inoculation with strain T4 dramatically raised the Cd enrichment coefficient of rice roots, respectively (**Table 1**) (*P* < 0.05). There was no significant change in the Cd transfer coefficient from roots to shoots of rice plants treated with either strain compared with CK (**Table 1**) (*P* < 0.05). These findings demonstrate that strain R3 inoculation mainly reduces the absorption of Cd by rice roots, while strain T4 inoculation mostly boosts rice roots’ ability to absorb Cd. Therefore, the root system is the key compartment influencing Cd absorption in rice plants.

### 3.2 Overall changes in the microbial community of rice roots under the action of endophytic bacteria

To further analyze the underlying mechanism of the effects of inoculated strains on Cd uptake in rice roots, we analyzed the overall changes in the endophytic microbial community in rice roots of inoculated and uninoculated treatments. We obtained an average of 22,372 raw sequences per sample, the resampling depth of each sample was 10203 sequences, and the cluster was 587 OTUs.

**Figure 1:**
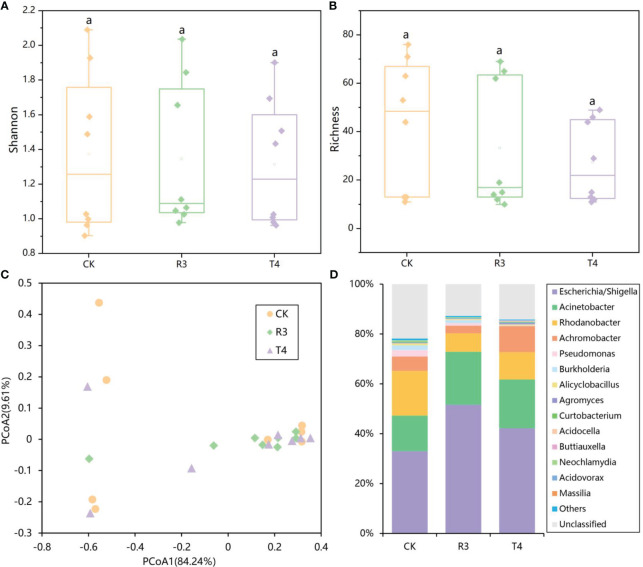
**(A)** Shannon-Wiener index, **(B)** Richness index, **(C)** 16S rRNA Gene Sequence Data using Principal Coordinates Analysis (PCoA) and **(D)** a detailed analysis of the relative abundance of endophytic populations at the genus level showing differences in the endophytic microbial community among rice roots with or without inoculation of test strains.

The rarefaction curves in **Figure S1** demonstrated that the sequencing depth was enough for the future analysis. Alpha-diversity analysis of the endophytic bacterial community in rice roots showed that the Shannon index, Richness index, Pielou evenness and Simpson index of the rice root microbial community after inoculation with strains R3 and T4 did not change significantly compared with the control (**Figures 1A, B**, **Figure S2**) (*P* < 0.05). PCoA (**Figure 1C**) analysis showed that the samples between the three treatments were intermixed. There was no significant change in bacterial community structure between the CK, R3, and T4 treatments were shown to be inconsequential by the different tests of MRPP, ANOSIM, and ADONIS (three nonparametric multivariate statistical tests) based on the Bray-Curtis matrix (**Table S2**). At the genus level, the most abundant genera in rice roots were *Escherichia/Shigella*, *Acinetobacter*, *Rhodanobacter*, *Achromobacter*, *Pseudomonas*, *Burkholderia*, *Alicyclobacillus*, *Agromyces* and *Curtobacterium*, which together accounted for more than 77% of the total abundance (**Figure 1D**). Compared with CK, the abundance of *Escherichia/Shigella* and *Acinetobacter* were increased in R3 treatment, while the numbers of *Rhodanobacter*, *Achromobacter* and *Pseudomonas* were decreased. The abundances of *Escherichia/Shigella*, *Acinetobacter* and *Achromobacter* were increased in the T4 treatment, while the abundance of *Rhodanobacter*, *Pseudomonas* and *Burkholderia* were decreased (**Table S3**) (*P* < 0.05). The results showed that inoculating R3 and T4 had no significant influence on microbial community diversity in rice plant roots, but did modify the relative abundances of their endophytic bacterial populations.

### 3.3 Correlation between rice plant-related indicators and root bacterial taxa

**Figure 2:**
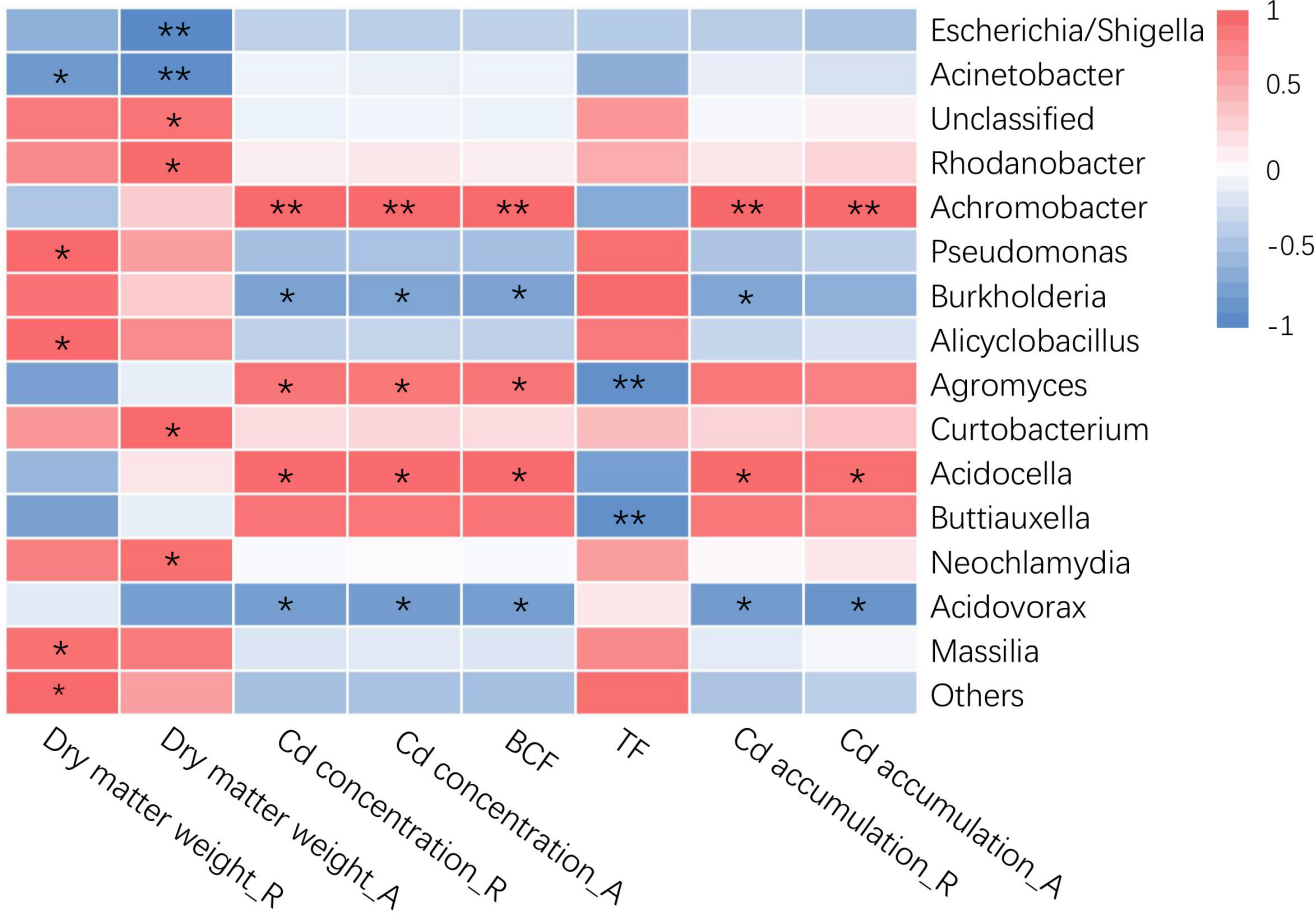
Correlations between root-specific enriched taxa (genus level) and plant variables of rice. “R” represents the root of rice, “A” represents the aboveground part of rice. The color represents the Pearson correlation coefficients (r). The asterisks in the grids show Pearson’s correlations that are significant: *, *P*<0.05; **, *P*<0.01.

The relative abundance of bacterial taxa in rice roots was substantially linked with some rice plant characteristics, according to Pearson’s correlation analysis (**Figure 2**). The results revealed that among the three sample groups, the bacterial groups connected with rice plant dry matter weight and Cd-related variables differed at the genus level. Rice plant dry matter weight was strongly negatively connected with *Escherichia/Shigella* and *Acinetobacter*, and considerably favorably correlated with *Rhodanobacter*, *Pseudomonas*, *Alicyclobacillus*, and *Massilia*. The Cd content and root Cd enrichment coefficient of rice plants were strongly favorably correlated with *Achromobacter*, *Agromyces* and *Acidocella*, and considerably negatively correlated with *Burkholderia* and *Acidovorax*. The Cd accumulation in rice plants was strongly favorably correlated with *Achromobacter* and *Acidocella*, and significantly negatively correlated with *Acidovorax*, and the Cd accumulation in rice roots was also negatively correlated with *Burkholderia*. The Cd transport coefficient from roots to aboveground parts of rice plants was negatively correlated with *Agromyces* and *Buttiauxella*. These results showed that rice plant Cd absorption is influenced by the structure and changes of the endophytic bacterial community in the roots, among them, more microorganism species and quantity (**Figure 2**, **Table S3**) exhibited a strong link with Cd absorption in rice roots, fewer microorganism kinds and numbers (**Figure 2**, **Table S3**) exhibited a significant link with the cadmium transfer coefficient from the root to the aboveground parts of rice. among them, the types and quantities of more flora (**Figure 2**, **Table S3**) were significantly correlated with Cd uptake by rice roots. The types and quantities of less flora (**Figure 2**, **Table S3**) were significantly correlated with the Cd transport coefficient from roots to aboveground parts in rice.

### 3.4 Topological characteristics of the microbial networks in rice roots under different strain treatments

**Figure 3:**
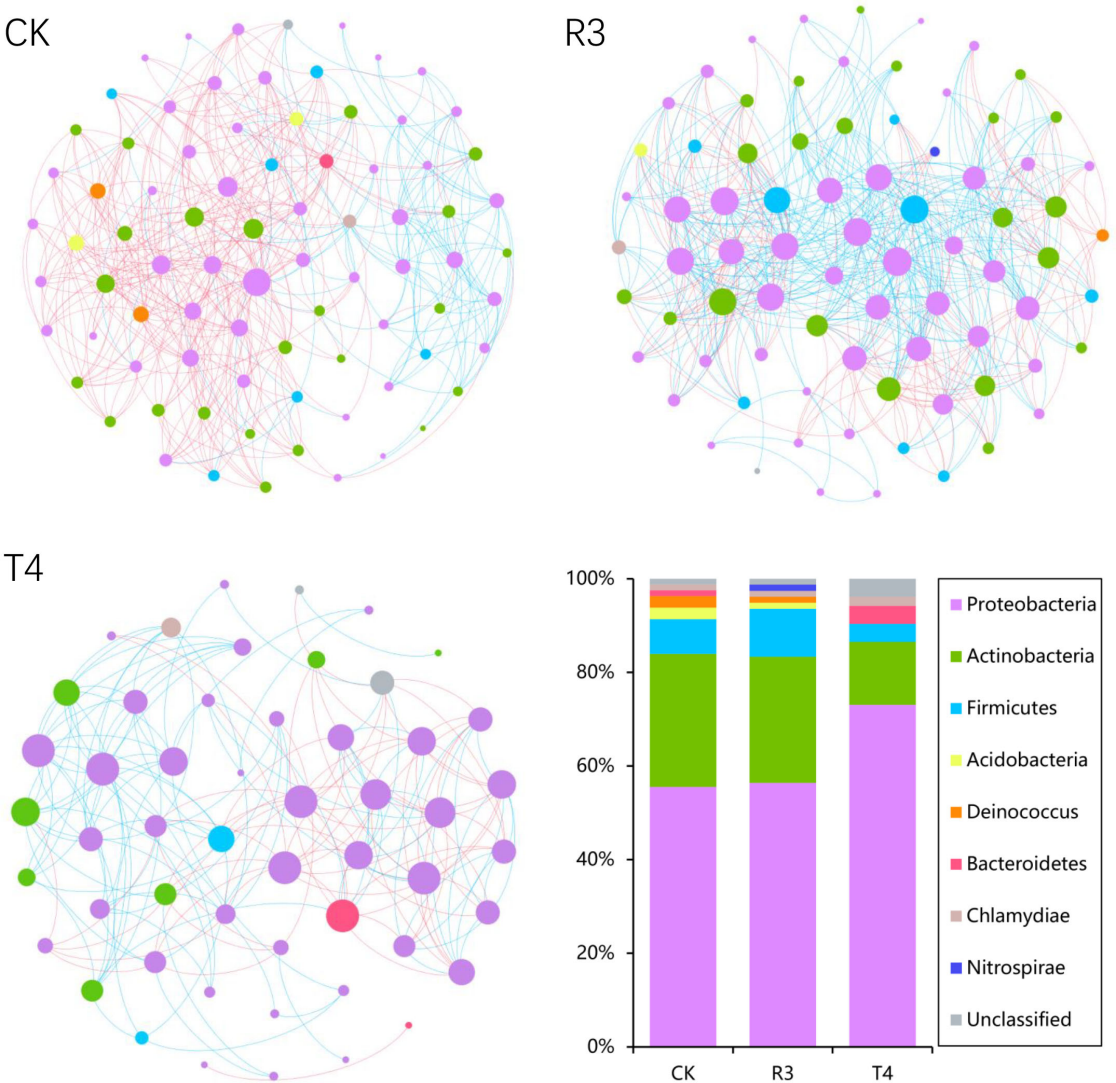
The co-occurrence networks for CK, R3 and T4, colored by phylum. A connection indicates a strong (Spearman’s |r|>0.8) and significant (*P <*0.01) correlation. Positive correlations are represented by blue lines, while negative correlations are represented by red lines. The node size represents the degree of OTUs.

**Figure 4:**
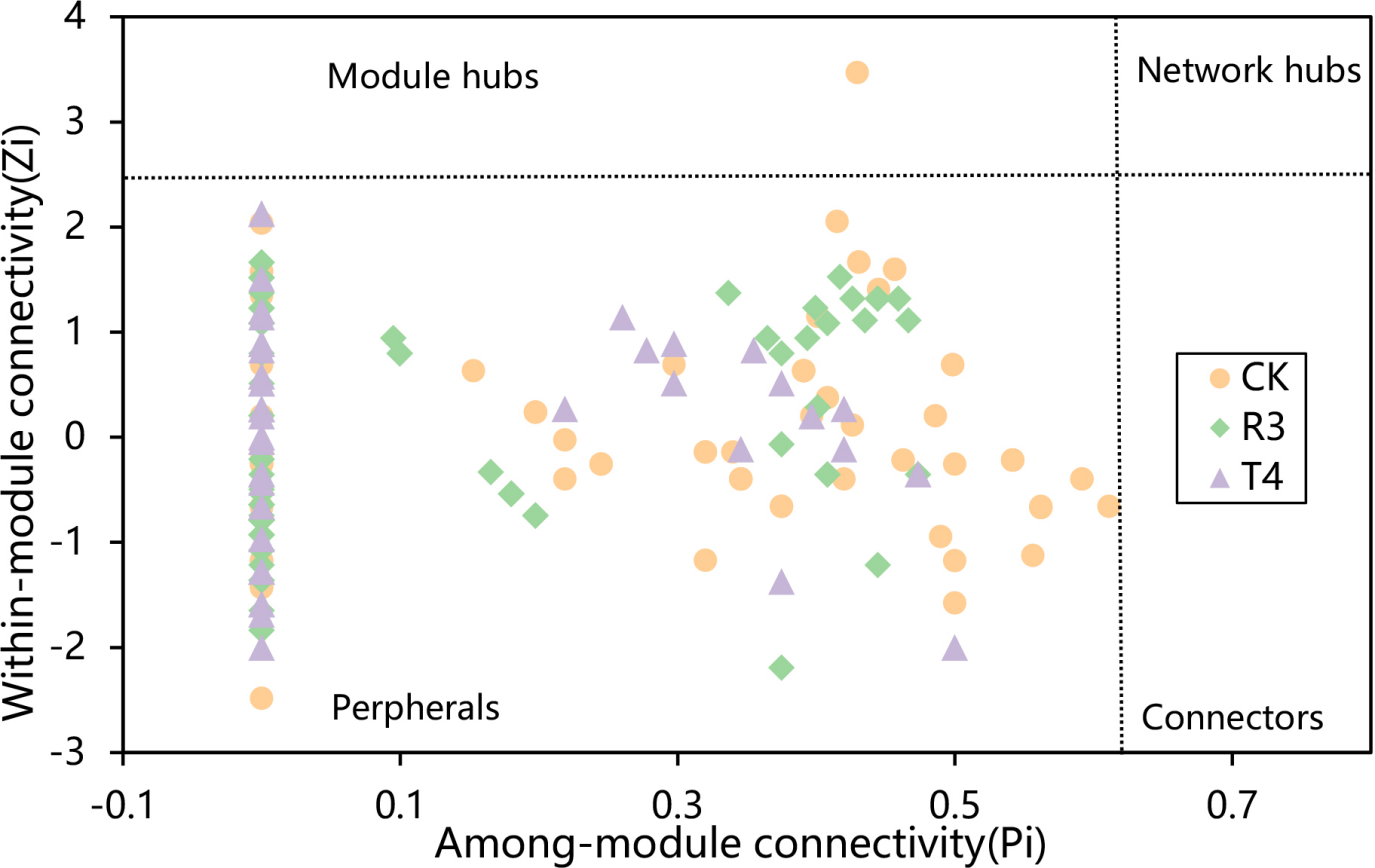
The distribution of OTUs in the three groups of samples was displayed using Zi-Pi plots based on their topological network roles. The Zi and Pi threshold values for classifying OTUs were 2.5 and 0.62, respectively.

To investigate whether the topological structure of interactions between endophytic bacteria in the roots of uninoculated and treated rice plants were different, we constructed endophytic bacterial community-related networks (**Figure 3**), which showed that endophytic bacterial interactions were different in response to the inoculated strains. The network inoculated with strain R3 had more links than CK, while the harmonic geodesic distance and average path distance were lower than CK. Compared with CK, the network inoculated with the T4 strain had fewer links and increased harmonic geodesic distance and average path distance (**Table S4**). Potential interactions within both the R3 and T4 treated endophytic bacterial communities had higher positive correlations compared with CK (**Figure 3**). Interestingly, only 5-8 phyla per treatment participated in the network, which may have been due to the dominance of these phyla in the respective endophytic bacterial communities, and further implied their critical roles in root function and ecological processes for rice. Compared with the CK interaction network, in the R3 treatment network, the frequency of *Acidobacteria* and *Deinococcus-Thermus* decreased, *Firmicutes* increased, and *Bacteroidetes* were absent, while *Nitrospirae* appeared. Compared with CK, in the T4 treatment network, the frequency of *Proteobacteria* and *Firmicutes* increased, while *Actinobacteria* decreased, and *Acidobacteria* and *Deinococcus-Thermus* were absent (**Figure 3**). Furthermore, we determined the topological role of each OTU in the endophytic bacterial network for each of the three treatment groups by RMT-based network analysis (**Figure 4**) and found that only one key taxon, OUT_471 (*Proteobacteria*), was detected in CK.

### 3.5 Association between network structure and Cd content in rice roots

**Figure 5:**
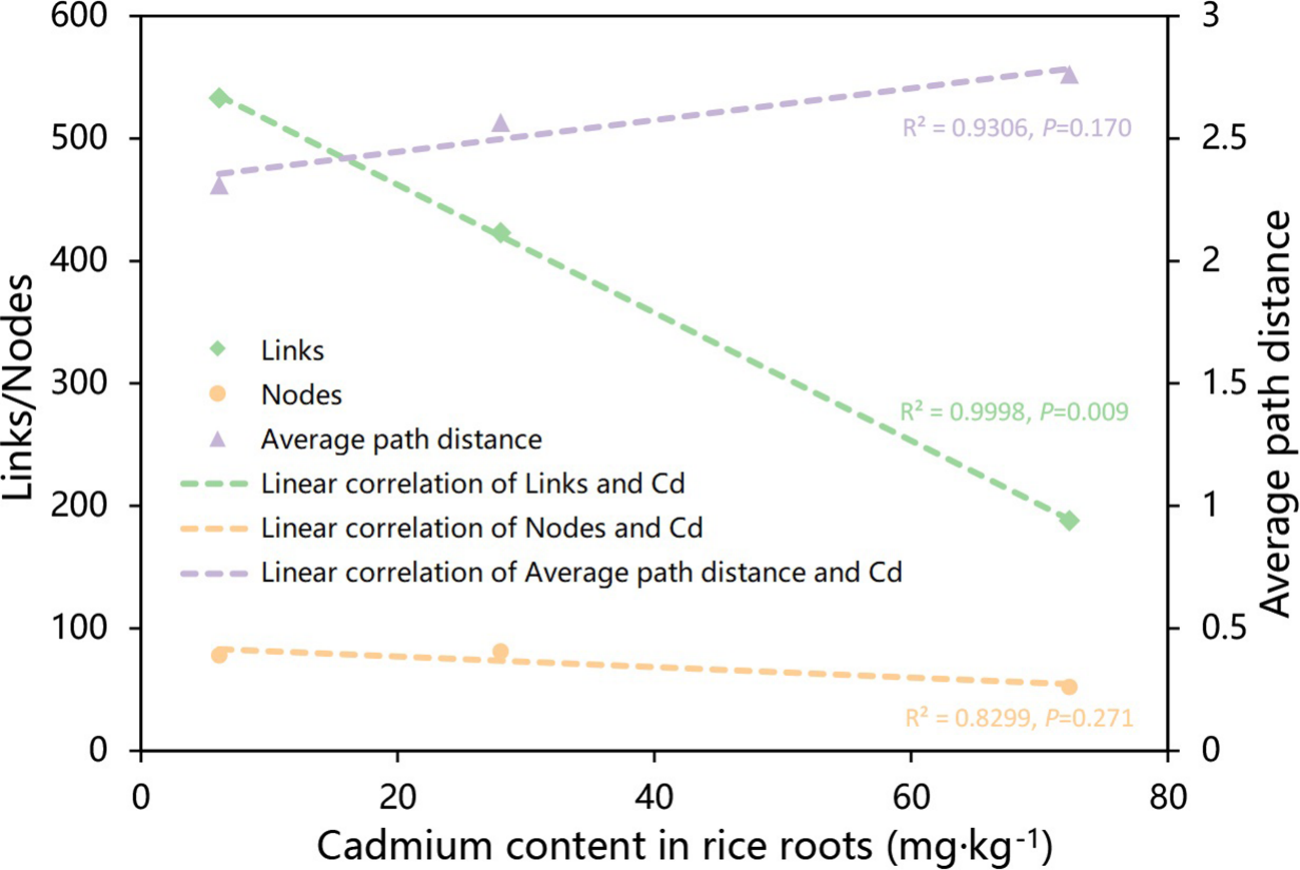
Plots of linear regression analysis illustrating the correlations between the nodes, links, and average path distance of the molecular ecological network and the Cd content of the rice. Linear regressions were detected for parameters that linked with rice Cd concentration considerably (*P* value < 0.05).

The network’s complexity may be represented by links, nodes, average path distance, and so on. A larger number of links and nodes, as well as a shorter average path distance, imply that a network is more sophisticated (Ye et al., 2012). According to Pearson correlation analysis, the number of links in the network was significantly inversely associated with Cd content in rice roots (*P*=0.009) (**Figure 5**), implying that lower Cd content in rice plants was correlated with more intricate networks in rice roots.

## 4 Discussion

This research evaluated the impact of two distinct bacterial strains on Cd buildup in rice plants and discovered that they had opposite effects. The inoculation of strain R3 exhibited a strong inhibitory impact on rice plant Cd absorption, and it could potentially be used as an exogenous additive to directly reduce the Cd content in rice plants. The rice plants’ ability to absorb Cd was greatly enhanced by strain T4 inoculation, and this strain was able to interact with heavy metal hyperaccumulators, allowing plants to efficiently enrich Cd in paddy soil. The Cd content of rice plants may be indirectly reduced by lowering the Cd content of the paddy soil by harvesting and disposing the Cd-enriched plants. Therefore, both strains have potential value in controlling Cd pollution in rice.

### 4.1 The overall impact of inoculated strains on rice growth and Cd uptake

As a heavy metal, Cd places stress on plant growth and development, and can also lead to plant death at high concentrations (Andresen and Küpper, 2013). Some microorganisms can mitigate the toxicity of Cd through biosorption, precipitation, complexation and chelation (Siripornadulsil and Siripornadulsil, 2013), meanwhile, there are also microorganisms that can promote plant growth through symbiotic interactions, auxin synthesis, and improved disease and stress resistance (Shan et al., 2020). In this study, the tested strains, R3 and T4, were endophytic bacteria screened from seeds of the rice variety Huanghuazhan, with functions such as tolerance to high Cd concentration, siderophore production, phosphorus dissolution and nitrogen fixation (**Table S5**). In the hydroponic pot experiment, after the rice roots were inoculated with the respective strains, it was discovered that there was no significant influence on rice growth and development. This may be because the nutrients in the growth solution were all in the form of ions, which could have been easily absorbed by the rice plants, and, as such, the phosphorus-solubilization, nitrogen fixation and other functions of the strains were not fully exerted, so no obvious growth-promoting effects were shown (**Table 1**).

Recent studies have found that microorganisms have a significant effect on Cd uptake by plants. For example, exogenous application of Bacillus *thuringiensis* X30 and Serratia *liquefaciens* CL-1 could significantly reduce soil available Cd content and rape Cd accumulation (Han et al., 2018). Similarly, *Bacillus strain* XT4 inoculation reduced soil available Cd content and Cd content in *Brassica rapa* (Zhang et al., 2021). However, it was discovered in another study that inoculating various strains of arbuscular mycorrhizal (AM) fungus considerably enhanced the absorption efficiency of Cd by *Sedum alfredii* Hance (Hu et al., 2013). The promotion or inhibition of Cd absorption from the environment by plants is mainly achieved by the adsorption, fixation or dissolution of Cd by various microorganisms, some of which modify the form of Cd, reducing its bioavailability, or fixing it with their own cells through adsorption, lowering the Cd content in the environment, as a result, lowering plant absorption of Cd (Li et al., 2018; Yuan et al., 2018; Wang et al., 2021), while other microorganisms can dissolve CdCO_3_ and solid Cd in the environment, increasing Cd’s environmental bioavailability (Yang et al., 2018), promoting the absorption of Cd by plants. Meanwhile, by fostering the growth of plant roots, some Plant Growth-Promoting Bacteria (PGPB) can enhance plants’ resistance to Cd stress and Cd buildup (Prapagdee et al., 2013). In order to verify whether the strains tested in the current study had an effect on the morphological transformation of Cd, we used *E. coli* as a positive control for a Cd^2+^ adsorption experiment. The findings demonstrated that strains R3 and T4 inoculation did not change the content of available Cd in the nutrient solution (**Figure S3**), therefore, it is first rejected that these two strains may have an impact on the Cd bioavailable in the environment. In our study, it was found that compared with CK, inoculation of the R3 strain could significantly lower the Cd concentration in each rice plant part that was examined for this study, while not significantly affecting the Cd transfer coefficient from the roots to the aboveground part, but significantly decreasing the Cd enrichment coefficient of roots to the environment (**Table 1**). It showed that strain R3 could reduce the Cd level of every region of the rice plant, mostly through diminishing rice roots’ ability to absorption of Cd from the environment. Compared with CK, the inoculation with the T4 strain significantly increased Cd content in all parts of the rice plant. While the transport coefficient of Cd from the root to the aboveground part did not change significantly, the Cd enrichment ability of the root to the environment was greatly increased (**Table 1**), indicating that strain T4 increased Cd content in all parts of rice plants mainly by enhancing the Cd uptake ability of rice roots. Therefore, one of the major affecting the Cd content of rice plants is the capacity of rice roots to accumulate Cd from the environment.

### 4.2 Effects of bacterial community structure and interaction in rice roots on Cd accumulation

The accumulation of Cd in rice plants mainly depends on the availability ofCd in the environment (Zhu et al., 2016; Wang et al., 2019), rice variety (Chen et al., 2019), rice planting management systems (Honma et al., 2016; Spanu et al., 2018; Wang et al., 2020b), physiological and ecological conditions of the rice plants (He et al., 2008; Uraguchi et al., 2009; Bari et al., 2020), and the endophytic microbial community (Zhou et al., 2020; Su et al., 2021). The effective Cd content in the environment did not significantly change in this study, and the rice varieties and planting management conditions were all the same. Therefore, variations in the endophytic bacterial community of the rice roots across the various treatments may be the reason for differences in Cd accumulation in rice plants. Studies have shown that plants rely on useful interactions between roots and microbes to acquire nutrients, stimulate growth and development, and withstand abiotic and biotic stresses (Edwards et al., 2015), therefore, the root microbial community is important in Cd accumulation in rice. However, little is known about how to root microorganisms influence Cd buildup in rice plants.

In this research, when compared with CK, neither of the rice root endophytic bacterial communities treated with strains R3 or T4 showed significantly altered diversity (**Figures 1A–C**, **Table S2**), indicating that the diversity of endophytic bacterial communities in rice roots was not a key factor affecting Cd uptake by rice. This was consistent with a study where it was shown *Sedum plumbizincicola*’s accumulation of Cd and Zn to be unrelated to the diversity of the endophytic bacterial community (Hou et al., 2017). However, the relative abundance of different endophytic bacteria in the roots of rice inoculated with strains R3 and T4 changed significantly (**Figure 1D**). This may be due to the fact that exogenous strains enter the tissues of rice through the lenticels or wounds (Compant et al., 2005), disrupting the makeup of the original microbial community, and forming a new stable community structure after a certain period of interaction. Studies have shown that inoculation with specific bacteria can alter the microbial community of plant roots, thereby altering symbiotic relationships involved in the turnover of soil organic matter (Ibekwe et al., 2013). Thus, we speculate that the different relative abundances of specific bacterial taxa were one of the explanations for the discrepancies in Cd accumulation in the rice plants.

Previous research has revealed a significant link between the Cd level of rice roots and the endophytic bacterial taxa (Chu et al., 2021). We discovered that there was a strong link between the bacterial taxa in rice roots and the Cd concentration in rice plants using Pearson correlation analysis (**Figure 2**). The results revealed that the Cd concentration and root Cd enrichment coefficient of rice plants were strongly favorably connected with *Achromobacter*, *Agromyces*, and *Acidocella*, and considerably negatively linked with *Burkholderia* and *Acidovorax*. At present, other studies have found that these bacterial groups interact with Cd. For example, *Achromobacter* has a strong Cd adsorption action (Abyar et al., 2012); *Agromyces* has a dissolving action on Cd and can improve plant Cd absorption (Kuffner et al., 2008; De Maria et al., 2011); *Burkholderia* Y4 can limit Cd accumulation in rice by boosting nutrient absorption, resulting in a competitive impact (Wang et al., 2020a). The variations in the physiological functions and relative abundances of various bacterial groups may be one of the key explanations for the discrepancies in Cd accumulation in rice, and the interaction of specific bacterial groups with crops and Cd requires further study.

Interactions between microorganisms have important roles in plant health (Durán et al., 2018), including plant growth synergistic effects (Van der Heijden et al., 2015) and microbiological pathogen antagonistism (Santhanam et al., 2015), and the mutual competition and cooperation between microorganisms contributes greatly to the entire microbial community (Hassani et al., 2018). In our study, we found that, while the number of nodes in the microbial network of rice roots treated with strain R3 did not change much in comparison with CK, the number of links increased significantly, and the average path distance decreased. In comparison, the rice treated with strain T4 has considerably fewer nodes and links in its root microbial network, and the average path distance increased (**Figure 3**). This indicated that after inoculation with strain R3, endophytic bacteria in rice roots interacted more complexly and closely, however, the interaction between endophytic bacteria in rice roots became simpler following inoculation with strain T4. According to relevant research, complex networks can better adapt to environmental changes (Berry and Widder, 2014; Zheng et al., 2022) or can better resist the damage of soil diseases to plants (Yang et al., 2017). The average path distance and the number of links between network nodes were both connected with the amount of Cd present in rice plants, as can be shown in **Figure 5**, whereas the network’s size was not the main factor impacting Cd absorption in rice. Therefore, there was a significant link between the network’s intricacy and the Cd content in rice plants.

It’s interesting to note that either strain inoculation significantly increased the positive (positively correlated) interactions in the rice root network (**Figure 1**). Positive interactions tend to indicate complementation or cooperation between nodes, while negative (negatively correlated) interactions imply predation or competition. Compared with CK, the root endophytes of both treatments had more harmonious symbiotic patterns (Ye et al., 2012). We speculate that the two strains’ inoculation initially altered the relative abundance of some endophytic bacteria that interact with Cd in rice roots, and that these changes may cause rice Cd uptake to exhibit the two different effects, either inhibiting or promoting uptake, and that the greater number of positive interactions may lead to more synergy between endophytic bacteria to jointly inhibit or promote Cd absorption and transport, thus causing the significant differences in Cd content of rice plants treated with the inoculated strains. These results suggest that inoculation of R3 and T4 strains not only had a significant impact on the functional structure of rice plant root microbial endophytic communities, but also on their microbial network interactions, with changes in these functional structures and interactions being significantly related to Cd concentration in rice plants. Further research on the physiological and biochemical properties of rice roots and the expression of genes associated to Cd absorption and transport will help to reveal the respective molecular mechanisms in rice of Cd uptake inhibition or promotion by the R3 and T4 strains.

## 5 Conclusion

Our research analyzed the influence of endophytic bacteria inoculation on Cd content in rice plants, and explored the effect of endophytic microbial communities in rice roots on rice plant Cd accumulation. The results indicated that the two strains, R3 and T4, respectively, exhibited significant inhibitory and promotion effects on Cd uptake in rice, and that this significant difference was mainly due to the altered relative abundance of endophytic bacterial taxa and their interactions in the rice roots. The higher the relative abundance of *Agromyces*, *Achromobacter* and *Acidocella* in the roots of rice plants and the simpler the network structure, the higher the Cd content of rice plants, but the higher the relative abundance of *Burkholderia* and *Acidovorax* in the root endophytic communities, and the more complex the network structure, the lower the Cd content of rice plants. Our findings imply that modulating the composition and interaction of root endophytic bacteria can efficiently minimize Cd buildup in rice plants without adverse effects. This study offers a different strategy to guarantee the cultivation of crops safely in paddy fields polluted by Cd, and a new microecological criterion for evaluating the role of exogenously added strains in Cd-contaminated remediation.

## Data availability statement

The datasets presented in this study can be found in online repositories. The names of the repository/repositories and accession number(s) can be found in the article/**Supplementary Material**.

## Author contributions

PL: Data curation, writing-original draft preparation. JL: Writing-reviewing and editing, validation. ZX, YT, ZZ, ZL and RH: Data collation and verification. QW, HA and ZY: Writing guidance. All authors contributed to the article and approved the submitted version.
