# Supplementary material for: Community-based mechanisms underlying the root cadmium uptake regulated by Cd-tolerant strains in rice (Oryza sativa. L)
Source: Front Plant Sci. 2023 Aug 11;14:1196130. doi: 10.3389/fpls.2023.1196130 (PMC10450764; doi:10.3389/fpls.2023.1196130)
Supplement: Supplementary file 1 [file DataSheet_1.docx]

**Community-based mechanisms underlying the root cadmium uptake regulated by Cd-tolerant strains in rice (*Oryza sativa*. L)**

Peng Li^a^, Ziqin Xiong^a^, Yunhe Tian^a^, Zhongyi Zheng^a^, Zhixuan Liu^b^, Ruiwen Hu^a^, Qiming Wang^a^, Hejun Ao^a^, Zhenxie Yi^a^, Juan Li^a^*

* Corresponding author at: College of Agronomy, Hunan Agricultural University, Changsha, China

Email addresses: adalee619@163.com (Juan Li).

^a^ College of Agronomy, Hunan Agricultural University, Changsha, China, 410128

^b^ Hunan Rice Research Institute, Hunan Academy of Agricultural Sciences, Changsha, China, 410125

**SM: Materials and Methods**

Data processing and statistical analysis

The transportation factor (TF) is the ratio of the amount of Cd in the aboveground sections of the rice plant to the amount of Cd in the rice plant's root. The bioconcentration factor (BCF) is the ratio of the amount of Cd in various rice plant parts and the amount of Cd in the environment. The T-test with SPSS 19.0 was used to compare the differences in physicochemical parameters across treatments, all significance tests were two-sided, and *P* < 0.05 was deemed statistically significant. The data in this research are the mean ± standard error (SE) of eight replicates.

Using the cutadapt program (<https://github.com/marcelm/cutadapt/>), primers were removed from the raw sequences to provide paired-end clean reads following a quality check, the forward and reverse sequences were then combined using FLASH (Magoc and Salzberg 2011), using the UPARSE (Edgar 2013) clustered operational taxonomic units (OTUs) at 97% similarity and categorized them using the RDP classifier (Wang et al. 2007), with a minimum confidence estimate of 50%. All of the above processes were carried out using a Galaxy analytic platform (<http://zhoulab5.rccc.ou.edu/>) (Afgan et al. 2016). Follow-up Analysis, including alpha diversity, beta diversity and dissimilarity test were carried out in R v2.11.1 with the package vegan v1.11-3. Differences in bacterial community composition between treatments were tested using permutation multivariate analysis of variance (Adonis). The pertinence relation between the root endophytic bacterial population and other rice characteristics was evaluated using the Pearson test. The genetic correlation network was investigated using Random Matrix Theory (RMT)-based methods by the Molecular Ecological Network Analysis Pipeline (MENAP) at <http://ieg4.rccc.ou.edu/mena>. Gephi 0.9.1-Beta (Bastian, Heymann and Jacomy 2009) and Cytoscape 3.4.0 (Cline et al. 2007) are used by the network to visualize. The association between the Cd concentration in rice plants and its network topological characteristics was examined using linear regression testing. The original sequencing data is housed in the Sequence Read Archive (SRA) database of the National Center for Biotechnology Information (NCBI) with the project ID PRJNA847405.

**SM: Tables**

**Table S1** Effects of endophytes on cadmium accumulation in rice plants.

| Treatment | Root Cd accumulation/ug·plant^-1^ | Aboveground Cd accumulation/ug·plant^-1^ |
| --- | --- | --- |
| CK | 18.46±1.18b | 5.92±0.86b |
| R3 | 3.55±0.33c | 1.14±0.17c |
| T4 | 43.41±3.52a | 11.24±0.89a |

Mean values (± S.D., n = 8) with different letters are significantly different among CK，R3 and T4 treatments based on Duncan test (P < 0.05).

**Table S2** Dissimilarity test of endophytic bacterial community in rice roots based on three non-parametric tests.

| Treatment | MRPP | | ANOSIM | | PERMANOVA | |
| --- | --- | --- | --- | --- | --- | --- |
|  | Delta | *P* | R value | *P* | F value | *P* |
| CK vs. R3 | 0.487 | 0.251 | 0.023 | 0.262 | 1.623 | 0.229 |
| CK vs. T4 | 0.562 | 0.563 | -0.054 | 0.720 | 0.608 | 0.501 |
| R3 vs. T4 | 0.418 | 0.561 | -0.037 | 0.595 | 0.584 | 0.478 |

MRPP: multiple-response permutation procedure; ANOSIM: analysis of similarities; PERMANOVA: permutational multivariate analysis of variance.

**Table S3** Differences in relative abundance of major bacterial groups in rice roots at the genus level.

| Genus | CK | R3 | T4 |
| --- | --- | --- | --- |
| *Escherichia/Shigella* | 33.022 | 51.674 | 42.179 |
| *Acinetobacter* | 14.181 | 21.189 | 19.552 |
| Unclassified | 21.843 | 12.800 | 14.242 |
| *Rhodanobacter* | 18.019 | 7.408 | 10.998 |
| *Achromobacter* | 5.739 | 3.011 | 10.415 |
| *Pseudomonas* | 2.636 | 1.052 | 0.256 |
| *Burkholderia* | 1.903 | 1.237 | 0.080 |
| *Alicyclobacillus* | 0.783 | 0.462 | 0.397 |
| *Agromyces* | 0.311 | 0.338 | 0.652 |
| *Curtobacterium* | 0.439 | 0.181 | 0.295 |
| *Acidocella* | 0.178 | 0.134 | 0.330 |
| *Buttiauxella* | 0.007 | 0.023 | 0.235 |
| *Neochlamydia* | 0.167 | 0.025 | 0.058 |
| *Acidovorax* | 0.044 | 0.066 | 0.042 |
| *Massilia* | 0.091 | 0.005 | 0.009 |
| Others | 0.646 | 0.393 | 0.262 |

**Table S4** Topological properties of the networks

| Root | Network index | CK | R3 | T4 |
| --- | --- | --- | --- | --- |
| Empirical networks | Similarity threshold | 0.910 | 0.910 | 0.910 |
|  | Total nodes | 81 | 78 | 52 |
|  | Total links | 423 | 533 | 188 |
|  | R^2^ of power law | 0.023 | 0.075 | 0.001 |
|  | Positive links (%) | 27.90 | 66.79 | 61.17 |
|  | Average connectivity | 1.000 | 0.878 | 0.925 |
|  | Average path distance | 2.565 | 2.310 | 2.762 |
|  | Harmonic geodesic distance | 2.133 | 1.902 | 2.188 |
|  | Average clustering coefficient | 0.265 | 0.529 | 0.313 |
|  | Modularity (No. of modules) | 0.389(3) | 0.290(3) | 0.379(3) |

**Table S5** Identification of physiological functions of tested strains

| Strain | Siderophore production capacity | Solubilizing phosphate ability | Nitrogen fixation function | Tolerant to cadmium concentration  /1 mmol·L^-1^ | Tolerant to cadmium concentration  /4 mmol·L^-1^ | IAA production function |
| --- | --- | --- | --- | --- | --- | --- |
| R3 | 1.20 | 1.20 | √ | ++ | + | √ |
| T4 | 1.40 | 1.23 | √ | ++ | + | × |

Note: "√" means yes, "×" means no, "-" means no colony growth, "+" means colonies appear, "++" means more colony.

**SM: Figures**


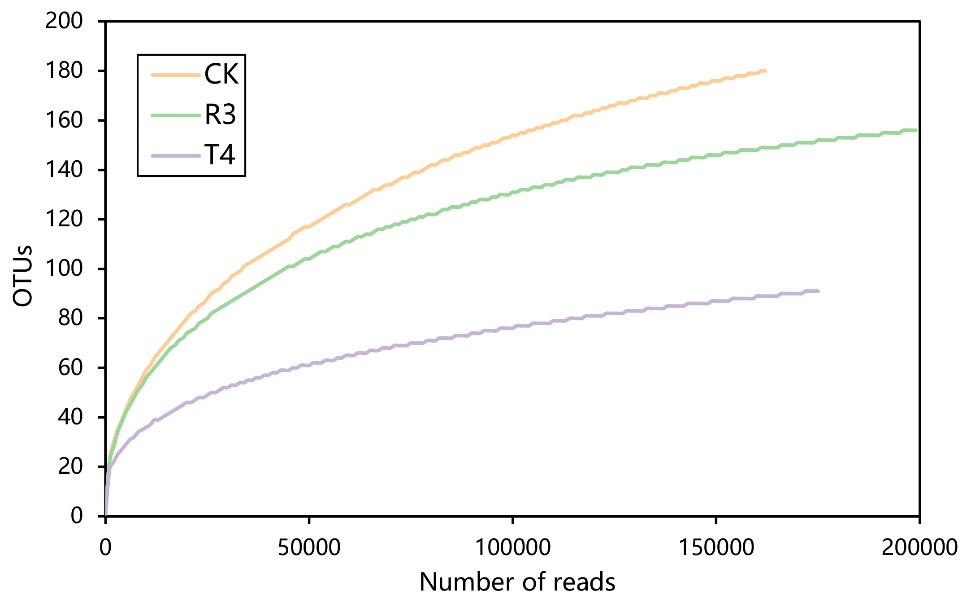


**Fig.S1.** Rarefaction curve of 16S rRNA gene sequencing among three treatment groups


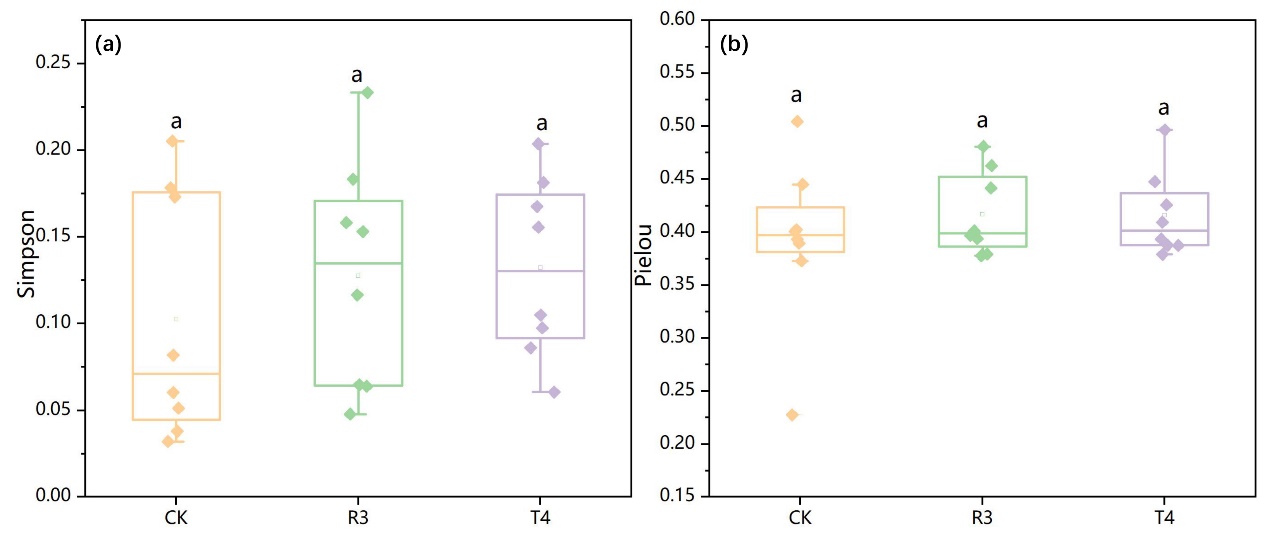


**Fig.S2.** Alpha-diversity of the three treatments. (a) Simpson-Wiener index; (b) Pielou Evenness


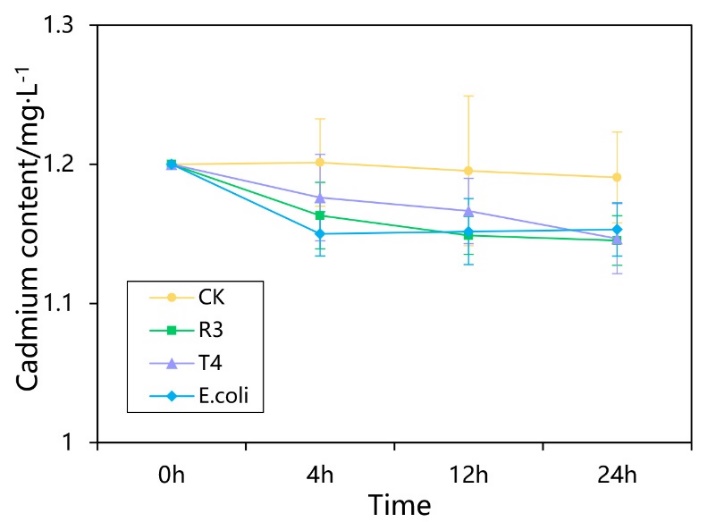


**Fig.S3.** Cadmium content in nutrient solution inoculated with different strains.

**References**

Afgan, E., Baker, D., Van Den Beek, M., Blankenberg, D., Bouvier, D., Čech, M., Chilton, J., Clements, D., Coraor, N., Eberhard, C., Grüning, B., Guerler, A., Hillman Jackson, J., Von Kuster, G., Rasche, E., Soranzo, N., Turaga, N., Taylor, J., Nekrutenko, A. and Goecks, J. (2016). The Galaxy platform for accessible, reproducible and collaborative biomedical analyses: 2016 update. *Nucleic Acids Research,* 44**,** W3-W10.

Bastian, M., Heymann, S. and Jacomy, M. (2009). Gephi: An Open Source Software for Exploring and Manipulating Networks.

Cline, M. S., Smoot, M., Cerami, E., Kuchinsky, A., Landys, N., Workman, C., Christmas, R., Avilacampilo, I., Creech, M. and Gross, B. (2007). Integration of biological networks and gene expression data using Cytoscape.

Edgar, R. C. (2013). UPARSE: highly accurate OTU sequences from microbial amplicon reads. *Nature Methods,* 10**,** 996-998.

Magoc, T. and Salzberg, S. L. (2011). FLASH: fast length adjustment of short reads to improve genome assemblies. *Bioinformatics,* 27**,** 2957-2963.

Wang, Q., Garrity, G. M., Tiedje, J. M. and Cole, J. R. (2007). Naïve Bayesian Classifier for Rapid Assignment of rRNA Sequences into the New Bacterial Taxonomy. *Applied and Environmental Microbiology,* 73**,** 5261-5267.
